# Simulating the extraction of signal parameters and spectrograms for non-stationary NMR signals

**DOI:** 10.5194/mr-7-125-2026

**Published:** 2026-09-14

**Authors:** Jixin Chen

**Affiliations:** 1 Department of Chemistry and Biochemistry, Nanoscale & Quantum Phenomena Institute, Ohio University, Athens, Ohio 45701, USA

## Abstract

Free induction decay (FID) signals in nuclear magnetic resonance (NMR) spectroscopy are commonly analyzed using the fast Fourier transform (FFT). This simulation study evaluates complementary methods for short or non-stationary FID signals in which linewidth, dephasing, or decay behavior changes during acquisition. Simulated FIDs containing exponentially damped sinusoids, Gaussian-distributed frequencies, and time-dependent broadening were analyzed using FFT, short-time Fourier transform (STFT), wavelet transform, and direct nonlinear time-domain fitting. Simulations of finite collection windows show that point-sampled FIDs can approximate window-integrated signals after amplitude and phase correction when the collection window is sufficiently sharp and reproducible. FFT remains the fastest and most reliable approach for stationary, sufficiently long, high-signal-to-noise signals. Zero filling improves spectral interpolation but does not recover frequency resolution lost through short acquisition. STFT and wavelet methods reveal time-dependent frequency and decay behavior, although with reduced frequency resolution. Direct time-domain fitting can estimate amplitudes, frequencies, phases, and decay constants from very short, relatively simple FID segments, but its reliability depends on model selection, initial estimates, the number of signal components, and avoidance of local minima. These methods therefore serve complementary roles in analyzing stationary and non-stationary NMR signals.

## 1 Introduction

Nuclear magnetic resonance (NMR) is widely used in chemistry, materials science, and life science. Solid-state NMR is one of the major spectroscopies used in surface chemistry and catalysis (Hamers et al., 2017; Weidkamp et al., 2004; Zhang et al., 2014). NMR's free induction decay (FID) signals are sampled in the time domain and are usually analyzed by the fast Fourier transformation (FFT) method (Hagaman et al., 1997; Esvan and Zeinyeh, 2020; van Beek, 2007; Orekhov et al., 2023; Stern et al., 2007; Hirakawa et al., 2019; Hulse, 2023). It converts the discrete signal collected with fixed time intervals from the time domain to the more intuitive frequency domain signal (Esvan and Zeinyeh, 2020; Kalstabakken and Harned, 2013; Orekhov et al., 2023). This analysis is done either manually or automatically (van Beek, 2007; Esvan and Zeinyeh, 2020; Hulse, 2023; Wu et al., 2024). These frequency signals are then correlated with the chemical environment of the atomic nucleus of interest to provide structural information about the molecules. The analysis often averages the dephasing signal of the decaying rotation of the atomic magnetization vectors. Because different atoms decay/damp differently and have different peak-broadening mechanisms, it is often difficult to correlate with the true amplitude of the signal that is proportional to the concentration of the corresponding atom. The traditional method is to simulate the chemical shifts using quantum calculations and then assign the peak intensities and shifts to different signals with a guessed molecular structure (Bothner-By and Naar-Colin, 1962). Advanced 2D NMR techniques such as COSY and DOSY have been developed to obtain time-evolved information (Hagaman et al., 1997; Nicolay et al., 2001; Sacristán-Martín et al., 2025).

For a given discrete dataset, the time-domain and Fourier-domain representations contain equivalent information in principle; the practical distinction concerns how easily and robustly particular parameters can be extracted. An alternative analysis approach is direct nonlinear time-domain fitting of parametrized damped sinusoidal models using least-squares or related objective functions (Castellano and Bothner-By, 1964; Montigny et al., 1990; Nishiyama and Mita, 1989; Vanhuffel et al., 1994). Nonlinear least squares gradient descent methods such as Gauss-Newton, and Levenberg–Marquardt algorithm have been used to fit NMR FID data. Within an assumed parametric model, direct time-domain fitting can provide more explicit estimates of phase, decay constants, initial amplitudes, and frequencies than are obtained from routine inspection of the Fourier spectrum. These estimates do not represent additional information beyond that contained in the measured FID, but they may be more directly accessible through model fitting. The initial amplitudes and peak areas are particularly useful to correctly quantify the atomic concentrations, and the decay lifetime helps find correlations between atoms, complementary to the traditional quantum simulation-assisted analysis and 2D NMR methods. We recently developed a jump-chain fitting algorithm (JCFit) that has shown some advantages in fitting damping wave functions compared to gradient descent methods (Chen, 2024). It searches exponentially apart from the original guesses of the parameters and skips the gradient calculation, thus making it more robust to the large number of local minima of a wave fitting project.

Artificial neural networks (ANN) have been used to fit the NMR FID data since the 1990s and have become very attractive recently (Hansen, 2019; Hiltunen et al., 1995; Karunanithy and Hansen, 2021; Kern et al., 2020; Lee et al., 2020; Li et al., 2021, 2023). ANN has been used to fit the frequency domain chemical shift NMR data or other spectra with convoluted Gaussian or Lorentzian peaks. The frequency domain peak fitting is to decouple overlapped peaks due to peak broadening, and the time-domain FID fitting is to obtain the chemical shift peaks with corrected peak heights and phases. While pattern recognition of chemical shift peaks and direct analysis of FID seems feasible, neural networks require high-quality simulated data to mimic the complicated collection conditions in experiments (Kern et al., 2020; Lee et al., 2020).

In this study, simulated FID signals are analyzed using FFT, STFT, wavelet analysis, and direct nonlinear time-domain fitting to evaluate their complementary roles and limitations. This study does not propose a new Fourier, wavelet, or nonlinear-fitting theory. Instead, it provides a simulation-based evaluation of the complementary roles of these methods for stationary, very short, and non-stationary NMR FIDs. The analysis focuses on dynamically broadened signals, the approximation of finite collection-window measurements by point sampling, and the potential and limitations of JCFit for estimating parameters from very short, relatively simple FID segments.

## 2 Methods

All simulations and analyses are done using MATLAB R2024a on a Lenovo laptop with an Intel i7-13800H CPU and 32 GB of memory. Parallel computation using 4 cores is sometimes introduced to slightly speed up the simulations. The source code is available online at GitHub https://github.com/nkchenjx/jcNMR (last access: 25 August 2026).

NMR FID signals are simulated using the chemical shifts of peaks. Three different peak shapes are used. (1) Single pure frequency with an exponential damping. (2) Peaks with Gaussian distributed frequencies with an exponential damping. (3) Peaks with Gaussian distributed frequencies that are broadening over time with an exponential damping. A summed chemical shift over all range of interests over time is constructed from these parameters, showing a damping chemical shift over time. The shift step is set to be 0.001 ppm, and the window is set to be -10 to 10 ppm to simulate ^1^HNMR.

The frequencies of the peaks are calculated from the chemical shifts ( δ ppm) of the peaks and the reference frequency $f_{\mathrm{ref}}$ ,

1 $$f_{\mathrm{peak}}\;(\mathrm{Hz})=f_{\mathrm{ref}}\delta_{\mathrm{peak}}\times 10^{-6}+f_{\mathrm{ref}}$$

e.g., for a reference frequency of TMS at $f_{\mathrm{ref}}=500\times 10^{6}$ Hz (default), and δ chemical shift (ppm), the frequency of an FID signal at a chemical shift $\delta_{\mathrm{peak}}$ centered at 1 ppm will have a center frequency of 500 000 500 Hz.

The FID signal of each data point at time t is numerically simulated from stepwise frequencies using the following equation,

2 $$I(t)=\sum_{\mathrm{peak}}\sum_{f}A_{f}e^{i\left(2\pi ft+\phi \right)}e^{-t/\tau }$$

Where $A_{f}$ is the amplitude of the signal at the frequency f , i is the imaginary unit $\sqrt{-1}$ , ϕ is the initial phase shift, and τ is the lifetime of the damping of each peak. The spacing step of the numerical frequency summation is chosen to be significantly smaller than the width of the peak for reasonable convergence to integrations. This equation is a combination of damping cosine and sine wave functions in real and imaginary dimensions. Positive imaginary terms fix the rotation to be clockwise and use -i if one wants anticlockwise, or combine both if needed. The anticlockwise signal at 1 ppm after clockwise FFT will be mirrored with 0 ppm and show up at -1 ppm.

The time sequence is selected at a sampling step $T_{s}$ such that the signal at the reference frequency is weak or invisible.

3 $$f_{\mathrm{ref}}T_{s}=2n\pi$$

Where n is an arbitrary integer of choice, or a random duration with a filtration of the reference frequency. The default $T_{s}$ is set to 0.0001 s and the number of data points is set to 2048 if not mentioned. The FID signal represents the difference between the frequency signal of a sample and the reference frequency because $T_{s}$ is chosen (Eq. 3) to cancel the signal from the reference frequency (Eq. 1). i.e.,

4 $$I(t)=\sum_{\mathrm{peak}}\sum_{\delta }Ae^{i\left(2\pi f_{\mathrm{ref}}\delta_{\mathrm{peak}}\times 10^{-6}t+\phi \right)-t/\tau }$$

Both time and signal noises are added to this signal to simulate an NMR measurement. The default time noise is 10 ps, and the default signal noise is set to be Gaussian with a standard deviation of 0.001.

Chemical shift peak broadening in the simulation window 0 ± 10 ppm is simulated using a discrete Gaussian function,

5 $$y=\frac{A}{\sqrt{2\pi }\sigma }\Delta \delta e^{-\frac{(x-\delta_{c}{)}^{2}}{2\sigma^{2}}}$$

Where A is the total peak area, assumed to be proportional to the concentration of the atoms in the molecule, σ is the standard deviation of the Gaussian peak, Δδ=0.001 (default) is the simulation step size of the chemical shift, x is the independent variable chemical shift with a step size Δδ , and $\delta_{c}$ is the center of the Gaussian peak.

The broadening of the Gaussian peak width over time is simulated to be

6 $$\sigma_{t}=\sigma_{0}+\sqrt{2Bt}$$

Where $\sigma_{t}$ and $\sigma_{0}$ are the standard deviation of the initial Gaussian chemical shift peaks at time t and zero, respectively, and B is the broadening rate, representing the effective diffusion coefficient with unit ppm^2^ s^−1^, which is correlated with the true diffusion constant of the molecule in the solution.

The simulated FID time sequences are used in further analysis, including discrete Fast Fourier transform (FFT) and direct nonlinear time-domain fitting. FFT converts simulated and experimental FID signals to chemical shift signals δ by,

7fkHz=∑n=1NIne-i2πfk(n-1)Ts8δkppm=fkfref×106

Where $f_{k}$ is the k th frequency of interest, n is the n th FID data point in the time sequence with time interval $T_{s}$ and the total number of data points N , $I_{n}$ is the FID signal, i is the imaginary unit, and $f_{\mathrm{ref}}$ is the reference frequency in Hz.

The real and imaginary components of the signal carry phase information and are distorted in the frequency domain. These peaks have Lorentzian line shapes on top of their simulated Gaussian broadening. FFT of only real signals or only imaginary signals produces peaks with mirror chemical shifts of the signals with respect to 0 ppm. Thus, it could be better to use the complex signal for FFT.

Direct nonlinear time-domain fitting is done using a published algorithm JCFit, whose detailed procedure has been explained before (Chen, 2023a, b, 2024). Briefly, each parameter in the wave damping model is scanned by simulation sequentially to find a fit to the simulated data with minimum sum squares of the residuals. The search sequence of the list of parameters is randomized at the beginning of each iteration.

Short-time Fourier transform (STFT), deep-learning STFT, and wavelet transform using simulated FID with 1024 data points. It simply places a selection window of the data points to run FFT and moves the window with fixed data points at a time. Piecewise data points from the FID time sequence are used to be convoluted with Fourier series or wavelet series with different frequencies to transform the 1D FID time domain data to 2D frequency domain spectra over discrete time, forming a spectrogram. An example of right-handed discrete wavelet transformation can be a 2D grid of frequency f and time t ,

9 $$Wlt(f,t)=\sum_{x=t-\frac{w}{2}}^{t+\frac{w}{2}}\text{FID}(x)e^{-\frac{(x-t{)}^{2}}{2\sigma^{2}}}e^{i(2\pi fx-\phi )}(9)$$

where w is the convolution window of the Gaussian wavelet, σ is the standard deviation of the wavelet, i is the imaginary unit, x is the time sequence of the convolution window, and ϕ is the original phase shift. For comparison, window width w is set to 256 data points, and each time step is 36 data points with 220 data points overlapping between adjacent time windows. Please check the source code for details.

## 3 Results and Discussion

### 3.1 Comparing simulation methods

**Figure 1:**
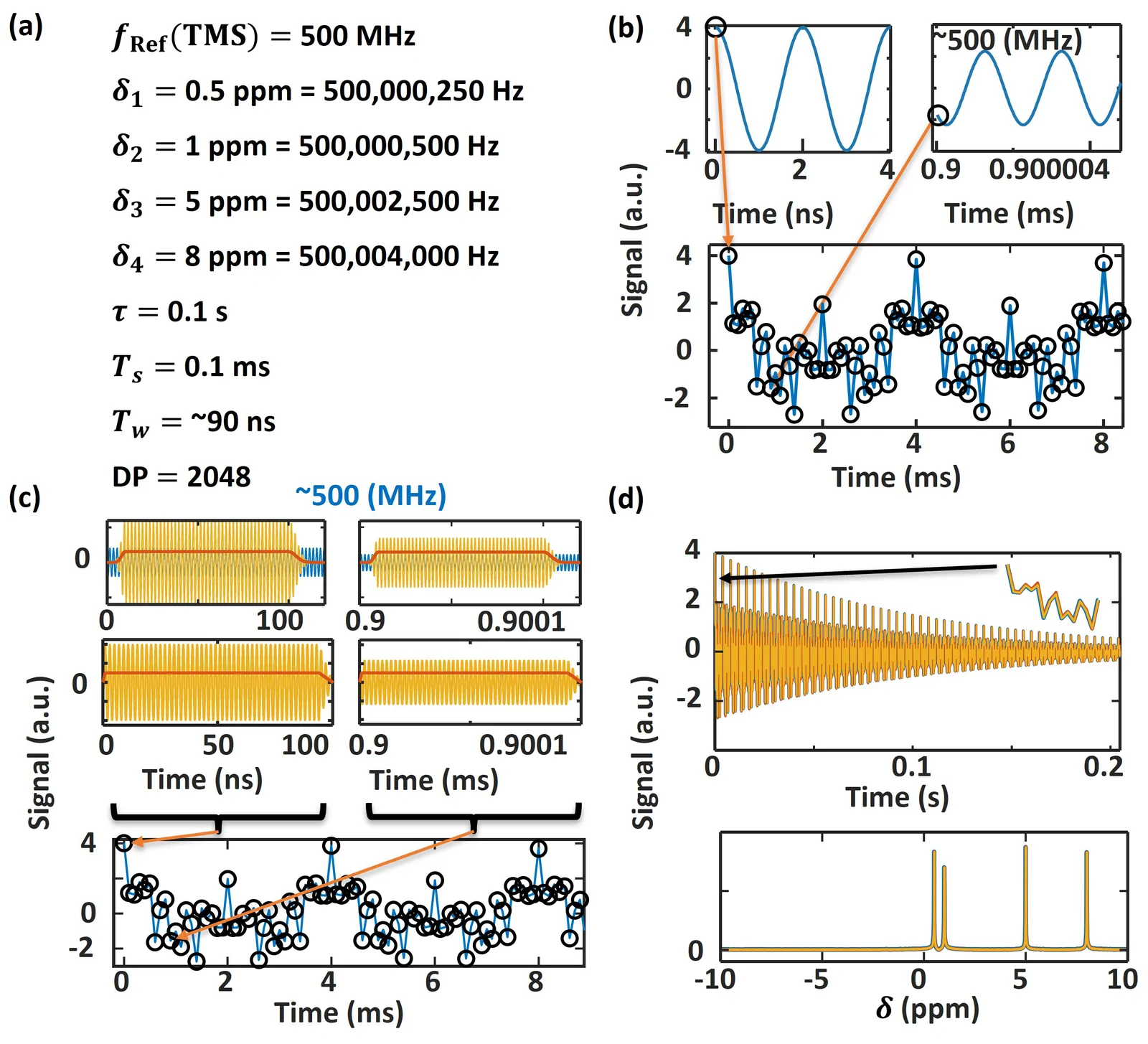
Simulating the effect of NMR data collecting parameters. **(a)** Simulation parameters of a ^1^HNMR FID signal with four frequencies, each having amplitude 1, phase shift 0 at time zero, and 0.1 s damping lifetime. **(b)** Scheme of using the initial combined signal at each time point as the FID signal. **(c)** Scheme of using the accumulated signal (summation) in each window as the signal. Two collection windows, one with Gaussian sides and the other with linear sides, are shown to have the same FID signal as in **(b)** after magnification and phase shifting. **(d)** Three FID simulations from **(b)**, **(c)** and their chemical shift peaks after FFT overlap with negligible differences after phase correction.

The actual collection of the FID signal during an experimental measurement is acquired at a time interval ( $T_{s}$ ) of each data point with an acquisition time window ( $T_{w}$ , typically $T_{w}\ll T_{s}$ ), i.e., the continuous waves are sparsely sampled. However, simulating the collection window is computationally costly, especially for signals with complicated peaks. Thus, a point sampling of the actual signal is preferred to simulate a lot of FID spectra. The effect of different collection windows is tested. No apparent difference other than phase shift and amplitude variation is observed under conditions simulating an experimental setting (Fig. 1). This null effect is reasonable because integrating a sine or cosine wavefunction is a complementary cosine or sine function, respectively, with the same frequency signals but different phases and amplitudes.

10 $$\begin{array}{rl} \text{FID}\left(t\right) & =\int_{t}^{t+t_{w}}e^{i\left(2\pi fx-\phi \right)}dx\\ & =\frac{1}{2\pi fi}[e^{i\left(2\pi f(t+t_{w})-\phi \right)}-e^{i\left(2\pi ft-\phi \right)}]\\ & =Ae^{i\left(2\pi ft-\phi^{\prime }-\frac{\pi }{2}\right)} \end{array}$$

Where t is the time when the corrector starts working, $t_{w}$ is a fixed-length-and-shape collection window, i is the imaginary unit $\sqrt{-1}$ , f is the true frequency of the signal, ϕ is the starting phase constant of the signal at time zero, x is time, A is a new constant, and $\phi^{\prime }$ is a new phase shift constant. This assumes that only the two side areas of the collection window contribute to the signal, and the middle part with full wave cycles is canceled. Thus, an ultra-sharp-edge rectangle collection window will give the same results as one-point data with phase and amplitude varied. A tunable edge slope and shape are simulated.

Fourier transform of the discrete FID signal regenerates a series of evenly distributed frequencies, including the original frequency,

11 $$\sum_{n=1}^{N}{\text{FID}}_{n}e^{-i2\pi f\left(n-1\right)T_{s}}=y[\left(f-f_{\mathrm{ref}}\right)+\frac{l}{T_{s}}]$$

Where l=1 , 2, 3,… and y is the signal in the frequency domain. The set of frequencies f between $f_{\mathrm{ref}}$ to $f_{\mathrm{ref}}+1/T_{s}$ (Hz) is used in experimental practices to calculate the chemical shifts.

12 $$\delta_{k}\;\left(\mathrm{ppm}\right)=\frac{f-f_{\mathrm{ref}}}{f_{\mathrm{ref}}}\times 10^{6}$$

Typical NMR collection windows of an NMR machine are trapezoids whose effects are simulated. The onset timing, end timing, and the edge slopes all affect the phase shift. The edge slope affects the amplitude, and the sharper the better (Fig. 1). For example, 500 MHz ^1^HNMR FID signals simulated using different methods are shown in Fig. 1. In addition to the amplitude noise determined by the FID collection precision, the quality of the signal is mainly determined by the accuracy of the starting time point and the repeatability of the collection window length. The time intervals are simulated to be <10 ps error in this example for >500 signal-to-noise ratio. The uniformity of magnification over the frequencies is correlated with the sharpness of the trapezoid's sides. In these simulations, a sharp <1 ns ramp time is needed for uniform amplification of signals for the chemical shifts of interest (0–10 ppm). As a reference, a 500 MHz rotational signal has a 2 ns wave cycle time.

Thus, the single-point value of the wave function at time t is used to simulate the FID signal instead of the integration of the wave signal in the correction window t to $t+t_{w}$ . The signal loss and phase shift due to collection windows can be simulated by tuning the amplitude and phase of the wave function. No obvious difference between simulations of one-point data collection and 90 ns data collection is observed, so one-point data collection is used in the following simulations. This choice significantly improves the simulation cost-efficiency.

**Figure 2:**
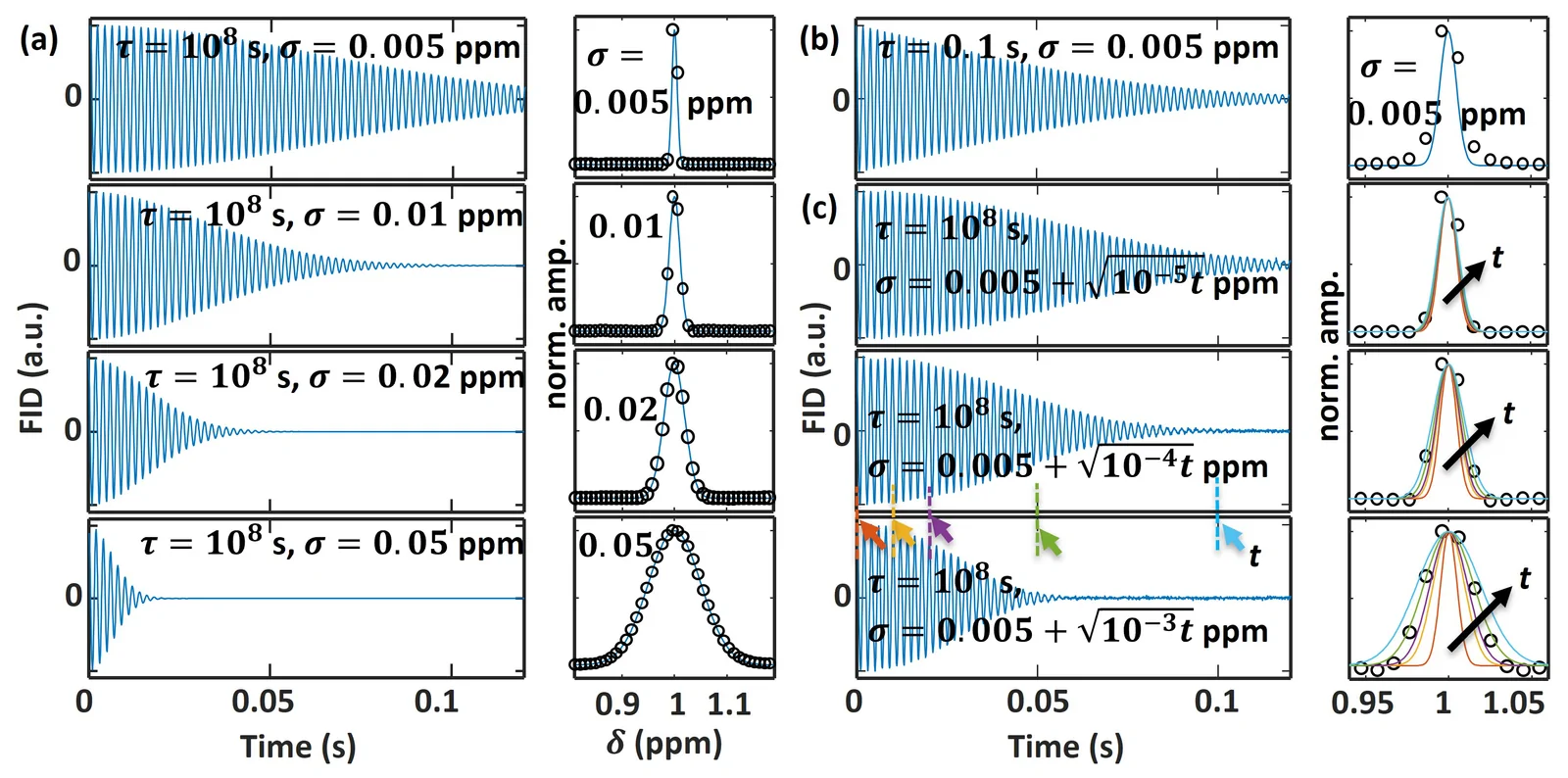
Simulated FIDs with chemical shift peak Gaussian broadened and their FFT peaks. TMS 500 MHz, δ=1 ppm with simulation resolution at step size 0.001 ppm, $T_{s}=0.1$ ms, data points 2048 one-point collection, and the rest of the parameters are shown in the figure. **(a)** No damping, only different Gaussian broadening for comparison. The Gaussian peaks for simulation are normalized by height with the overlay circles representing the normalized FFT results. **(b)** Adjusting the damping lifetime to have a comparable effect on the FID decay rate as the Gaussian peak broadens. **(c)** The effect on the FID decay of Gaussian peaks evolving broader over time.

FID signal of chemical shifts with Gaussian broadening and diffusion is then simulated (Fig. 2). Once a fixed peak broadening over time is introduced, both dephasing (interference) and damping control the decay speed of the FID signals (Fig. 2a, b). For each chemical shift peak, many signals with slightly broadened frequencies are first synchronized in phase, and then they decay due to damping. They also start to lose phase synchronization over time, and the signal cancels out due to interference. Thus, the FID signal with measurable amplitude is significantly shortened, dominated by the dephasing of the signals when the damping is negligibly small with a very large decay lifetime e.g. 10^8^ s, ∼3 years. (Fig. 2a). Peaks further apart in chemical shifts usually do not interfere with each other.

When a comparable damping lifetime is introduced, e.g. 0.1 s in Fig. 2b, the FID decays faster than no damping. Damping also adds additional broadening to FFT peaks. At this damping rate, the real part of the FFT signals with proper phase corrections has peaks slightly broader than the true values (Fig. 2b).

When the Gaussian chemical shift peaks are simulated to be dynamic and further broaden over time, the FID signal decays faster than the fixed-width Gaussian peak simulations due to the dephasing increases with the further broadening over time (Fig. 2c). When the damping is set to be negligible to simplify comparison, the real part of the FFT peak is consistent with the Gaussian peak width of the latter tail part of the FID signal with measurable amplitude. This consistency is expected because FFT reflects the average peak width of the useful signals.

Simulating 1000 random FIDs each with 1024 data points and tens of randomly evolving Gaussian chemical shift peaks with 0.001 ppm resolution takes ∼1 h, for a single CPU of a typical laptop or desktop with an Intel i7 CPU. Parallel computation using multiple CPUs or GPUs can significantly speed up such batch simulations.

### 3.2 Direct nonlinear time-domain fitting of NMR FID signals

**Figure 3:**
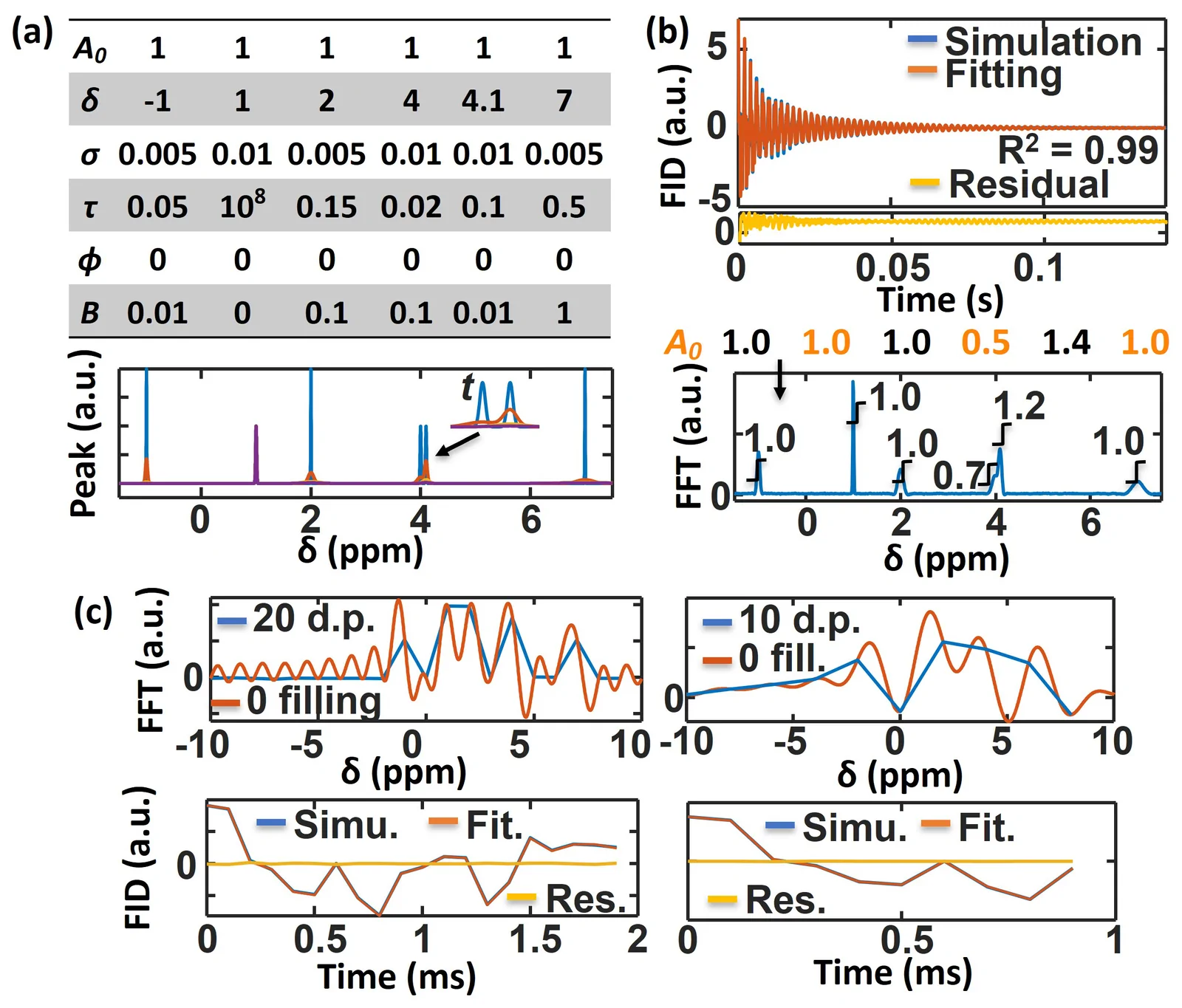
Direct nonlinear time-domain fitting of simulated FID signals. **(a)** Simulation parameters of 500 MHz ^1^HNMR. **(b)** Least squares fitting of simulated 2048 data point FID using single frequency; fitted $A_{0}$ ; and real part of the FFT of FID with integrated area shown with error <±0.1 ppm except for the doublets at 4 ppm. **(c)** Simulations with 20 and 10 data points using the same parameters, adding some random initial phase shifts. The real part of FFTs, and the direct nonlinear time-domain fitting of the FID data are shown, which still yield an accuracy of chemical shift within 0.1 ppm except for the peaks at 4 ppm. 20 data points (500 Hz) are about one wave cycle of the peak at chemical shift ∼1 ppm.

The Jump-Chain Fitting (JCFit) algorithm we developed can fit FID using the initial guess obtained from the FFT results. For an example simulation of chemical shifts with fixed Gaussian width over time (Fig. 3a), JCFit using the simplest model, damping single frequency (no width, no broadening, and no initial phase shift) with initial guesses from the FFT results, can fit the FID with reasonable initial amplitudes with $R^{2}=0.99$ (Fig. 3b). For a well-posed problem that converges to a unique global optimum, the initial guesses would not affect the final solution; in practice, they strongly influence convergence and the solution obtained for non-convex or poorly conditioned problems. The amplitudes are similar to the FFT results, yet FFT is orders of magnitude faster than JCFit. Because no peak width and dephasing are fitted, the damping rates are off the true values. Initial phase shifts if simulated, can be correctly fitted. If the same model with wave interferences as the simulation is used during the fitting, JCFit has the potential to fit the parameters but will be unacceptably slow.

A potential advantage of parametric model fitting under strongly constrained conditions can be illustrated using very short simulated signals. For this simple simulation, JCFit fitted FIDs containing only 20 or 10 data points within a few seconds, whereas Fourier-domain peak estimates became poorly resolved, e.g., it takes a few seconds to fit 20 or 10 data points that are shorter than the wave cycle of the slowest frequency (21 data points for δ=1 ppm). FFT struggles to obtain enough resolution for data shorter than one cycle. The FFT of data with or without 0 added after the data to 2048 data points, is significantly less accurate than the FFT of longer data (Fig. 3c). Zero filling is commonly used in Fourier-domain NMR processing, (Comisarow and Melka, 1979) but it does not add information beyond that contained in the acquired data. For this simple simulation, the fitted chemical shifts and amplitudes of most isolated peaks agreed with those obtained from the longer FID within 1 % error (Fig. 3c). However, the doublet near 4 ppm remained unresolved, as it did in the Fourier analysis and fitting of the longer signal, and its fitted chemical shifts showed errors of approximately 0.2 ppm. When the observation window contains only a partial oscillation cycle, the corresponding Fourier-domain feature becomes broad and frequency estimation becomes poorly conditioned. Zero filling improves spectral interpolation but does not recover the resolution lost through short acquisition. Parametric time-domain fitting may nevertheless estimate a frequency under sufficiently accurate model assumptions and prior constraints, although such estimates are strongly model-dependent. For this simple, model-matched simulation, direct fitting produced more accurate parameter estimates than Fourier-domain peak estimation for most isolated components.

This result does not establish general superiority because the fitting performance depends strongly on model accuracy, signal complexity, spectral overlap, noise, and prior constraints. Direct nonlinear time-domain fitting of parametrized damped sinusoidal models may provide complementary estimates of frequencies, amplitudes, phases, and decay constants for very short and relatively simple FID signals. However, the fitted solution may be non-unique or poorly conditioned when the model contains many overlapping or weakly constrained components. Its reliability therefore depends strongly on the assumed number of signal components, the model form, parameter constraints, initial estimates, signal-to-noise ratio, and, where appropriate, regularization or model-selection criteria.

### 3.3 Short-time Fourier transformation, wavelet, and machine learning

FFT is sensitive to data length but does not need the whole FID signal for high-resolution frequency transformation. Short-time Fourier transformation (STFT) and wavelet transformation (WT) are natural extensions of the FID data analysis and have shown potential in NMR tuning (Hirakawa et al., 2019; Kim et al., 2015; Liu et al., 2016). STFT breaks the FID into pieces or uses a moving window to select the subsets of data for FFT analysis with/without zero filling and plots the frequency signals in a time sequence, which can be used to construct a frequency-time spectrogram. For example, apply FFT to FID data at 0–0.02, 0.01–0.03 s, etc., with a 20 ms time window. Spectrograms are commonly used for machine learning, such as sound pattern characterization. The piecewise FID signal decouples some peaks with different decay lifetimes, and the short sequence contains less random noise. Both destructive and constructive interference among peaks are observed. Wavelet transformation uses a similar idea but directly applies wave packets to move through the FID signals to extract the frequency signal that matches the wave packets' frequencies. It usually uses a shaped wave instead of a rectangular wave in the FFT. For example, moving convolution Gaussian wavelet with frequencies 1, 2, … Hz to the FID data over time. The Gaussian-windowed wavelet analysis used here provides a time–frequency representation of the FID and is not intended as an orthogonal basis expansion or as a method for fitting symmetric NMR line shapes. The resulting spectrogram should therefore not be interpreted as an exact reconstruction of the underlying spectral line shape. Deep learning STFT (DLSTFT) constructs piecewise frequency peaks for neural network training.

**Figure 4:**
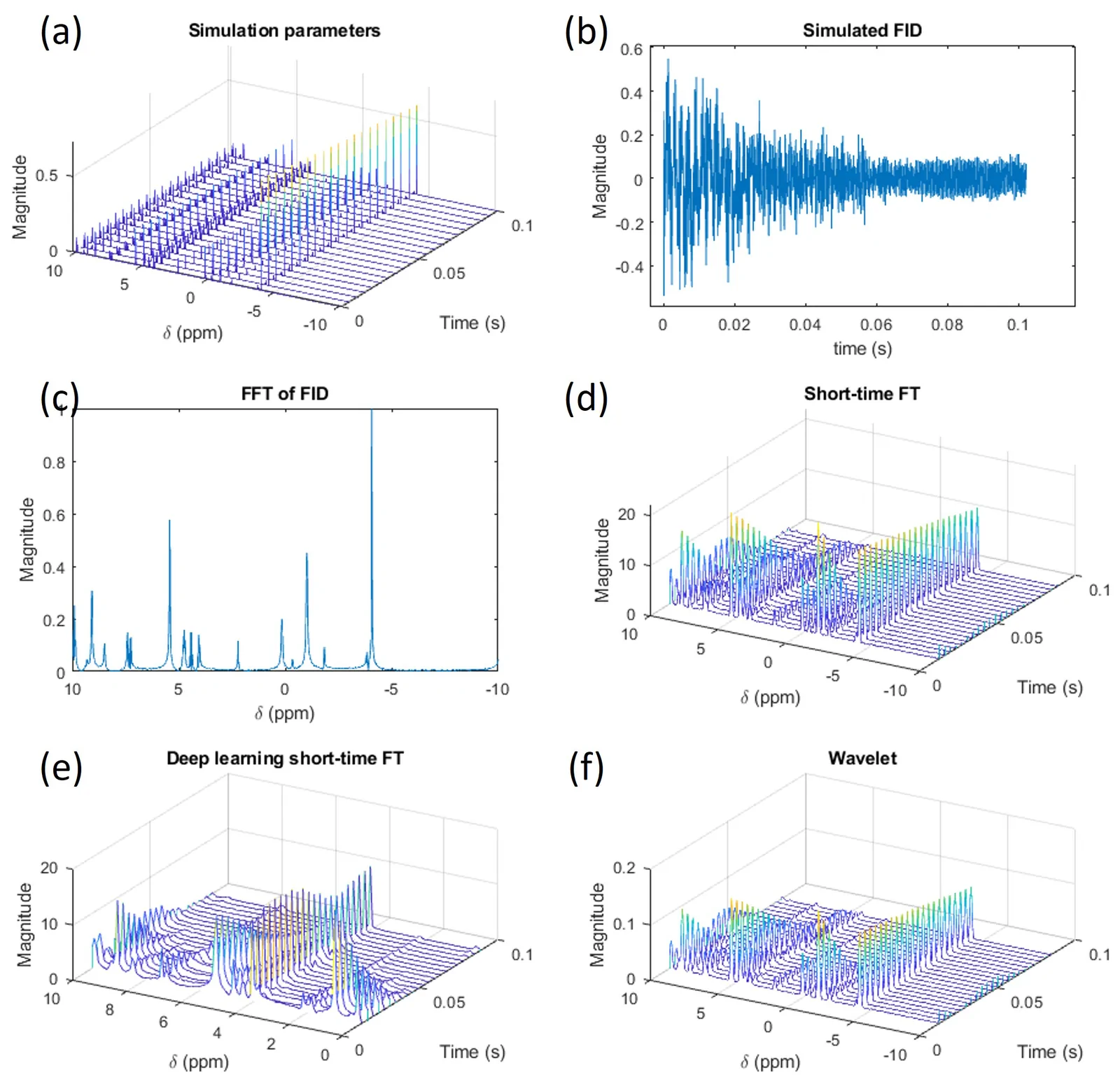
An example of 10 000 randomly simulated ^1^HNMR and analysis results. **(a)** Gaussian chemical shift peaks damping over time with no further broadening. **(b)** The real part of the simulated FID. Absolute values of **(c)** FFT, **(d)** STFT ( ∼0.02 s data per time step), **(e)** DLSTFT from MATLAB does not support complex input, so peaks are mirrored over the reference shift at zero, and **(f)** wavelet analysis of the simulated FID signals.

Short-time Fourier transform (STFT) of FID offers a dynamic view of the decay of frequencies over time and their interferences. Figure 4 shows a comparison of FFT and piecewise short-time FT methods in analyzing simulated ^1^HNMR FID signals. STFT, DLSTFT, and wavelet Fourier transform show similar dynamics of the chemical peak decay with dephasing and interference effects. All three methods reduce the frequency resolution compared to FFT as expected. These methods use shorter pieces while FFT uses the whole FID data set. STFT offers the best peak resolution using sharp-edged convolution wave windows. Gaussian wavelet FT using wave packets has “softer” window edges and shorter effective window sizes, thus, its frequency resolution is poorer than STFT with a similar window size. The Gaussian-windowed wavelet representation may reduce sensitivity to sharp window-edge effects, although it provides lower frequency resolution than STFT for the window sizes examined here. Among the methods tested, the MATLAB DLSTFT implementation produced the lowest frequency resolution and did not support complex-valued FID input in the software version used here.

## 4 Conclusion

This report compares simulation and analysis methods for NMR FID signals, including FFT for frequency-domain representation, STFT and wavelet analysis for time-frequency visualization, and direct nonlinear time-domain fitting for estimating parameters within an assumed signal model.Chemical shift peak frequencies can be used to simulate FID signals at collection time steps with intervals much longer than the wave cycles. However, simulating the broadening of these peaks reveals that the interference of different parts of the peak dampens the intensity of the FID signal and cannot be ignored. Therefore, the starting point signal of the data collection time step for each frequency is summed to obtain the apparent FID data point. The simulation frequency resolution should be much finer than the peak width of the chemical shifts.

Ideally, integrating the convolution of the collection window with the hidden FID signals would simulate the experimental data collection process, but this is computationally expensive. If the collection window edge is sharp enough, its effect can be relatively small.

FFT offers a very fast and accurate transformation of time domain FID signals to frequency-domain spectra and remains the preferred method for stationary, sufficiently long, high-signal-to-noise data. Mathematically, the discrete Fourier transform can also be viewed as an orthogonal least-squares projection onto the Fourier basis. When the acquisition duration is comparable to or shorter than one wave cycle, the resulting Fourier spectrum becomes intrinsically broad and poorly resolved because of the finite observation window. Zero filling can interpolate the discrete spectrum and improve its visual appearance or facilitate peak picking, but it does not add experimental information or recover the missing frequency resolution. For such short signals, direct nonlinear time-domain fitting of parametrized damped sinusoidal models may provide complementary estimates of frequencies, amplitudes, phases, and decay constants. However, this approach is challenging because the objective-function landscape can contain many local minima, and the fitted results depend strongly on the assumed number of signal components, the model form, the initial parameter estimates, and the signal-to-noise ratio. The recently developed jump-chain fitting algorithm searches a broader parameter range during each iteration and may reduce sensitivity to local minima compared with conventional gradient-based optimization. Although computationally expensive for long or complex FIDs, it can be useful for very short, relatively simple signals when the number of components is small or constrained by prior knowledge. Direct fitting becomes increasingly difficult for complex molecules with many overlapping and unknown components; in such cases, Fourier-domain analysis and prior chemical information can provide initial frequency estimates for subsequent model fitting to estimate parameters associated with non-stationary decay behavior. However, fully automatic and reliable determination of the number of signal components remains challenging.

Deep learning analysis of FID signals requires high-quality spectrograms. Simulated data are needed to prepare the training library for chemicals collected using different NMR machines and data collection conditions. The current comparison shows that STFT offers good frequency resolution in generating spectrograms from FID data. For FID with mixed chemicals, a potential challenge can be the complicated interference patterns on the spectrograms among peaks from different compounds. Nevertheless, using STFT, DLSTFT, or wavelet spectrograms to train artificial neural networks that can quickly classify chemicals is a promising future pursuit, analogous to classifying human speech.

## Data Availability

The source code of JCFit in MATLAB is also available on GitHub https://github.com/nkchenjx/JCFit (Chen, 2023b). The source code for generating the figures in this report is available at GitHub https://github.com/nkchenjx/jcNMR (Chen, 2026).
